# The Cheap Doctor

**Published:** 1902-01-11

**Authors:** 


### The Hospital

A Journal of

The Medical Sciences and Hospital Administration.

Vol. XXXI.—No. 798.] Edited by Sir Henry Burdett, K.C.B., and Solomon C. Smith, M.D., M.R.C.P. [January 11, 1902

### THE CHEAP DOCTOR.

It has often been said, and not altogether without reason, that members of the medical profession are a disunited flock; and certainly the history of the past gives us but few instances in which doctors have been able by combination to obtain for themselves any great advantage. Signs are not absent, however, which suggest that even the profession of medicine is not entirely unwilling to be "organised," and that backed up by certain special organisations which have arisen for the protection of medical interests, and supported by the decisions of the General Medical Council, certain groups of practitioners may before long endeavour to bring pressure upon their fellows in regard to details of their professional work which have hitherto been regarded as purely personal concerns. More especially is this likely to be the case in reference to fees. The old feeling that medicine is a noble profession is still strong within us. Most of us would probably scorn the idea of any association with trades unionism. A majority, even among those who have combined in association for mutual protection, would, we think, be prepared to maintain that if ever they interfere with the proceedings of a professional brother it is because of some ethical backsliding on his part. But the boundary line between medical ethics and medical fees is curiously thin, and no one can doubt that in some of the cases in which we hear of medical men being brought to book on questions of medical ethics, the real *casus belli*, the thing that has brought the matter home, has been something to do with fees. Lest anyone should say that this is not so, and that in speaking thus we are defaming our profession, we would refer to the celebrated Irvine case. In this case it was widely believed and maintained by medical men that the charge was one of advertising, and so fully did this represent the general feeling that the whole medical press with one accord raised a howl of indignation when Mr. Chamberlain in the House of Commons suggested trades unionism and hinted at fees being at the bottom of the matter. Yet, even in that very case, if we look at the actual wording of the charge—a charge which we may presume was formulated with the help of the combined acumen of the various persons concerned in the prosecution—we find that the fee question could not be kept out, and that the charge really was one of acquiescing in the issue of advertisements "inviting the public to consult him *at a reduced fee!*" At present the question of interfering with fees crops up mostly in connection with contract practice, but if organised effort should succeed in driving up contract fees, it will not be long before pressure will be brought to bear upon the lower grades of general practice, and, although we sympathise to the full with the proper aims of the great societies which are growing up within the profession for its protection, and with the object of fighting those aggressive organisations outside by which the independence of professional life is being threatened, we cannot but feel that the time is coming when a firm stand will have to be made to prevent these societies from interfering with the inalienable right of every practitioner to charge what he likes for his services. The thongs and scourges by which the doctors are to be driven to reform their ways are the dicta which the General Medical Council can be persuaded from time to time to pronounce as to what constitutes "infamous conduct in a professional respect," and the General Medical Council, not being always very wise, is apt to do things the consequences of which it does not see. It is hardly eighteen months since the Council, being asked to anathematise the medical aid associations, refused to go further than saying that it "strongly disapproves of medical practitioners associating themselves with medical aid associations which systematically practise canvassing and advertising for the purpose of procuring patients." It was then maintained that this resolution committed the Council to nothing. Its wording was entirely different from that adopted in regard to "covering" and the employment of unqualified assistants; many members of the Council looked upon it as a mere sop to the agitators, and it was openly spoken of as a "pious opinion" and nothing more. Yet we have already seen at least two prosecutions under this very resolution. What then is to prevent this pliant Council, which so little knows its own mind, from passing a resolution to the effect that it "strongly disapproves" of a sixpenny fee? It would be quite as easy to prove the immorality of the sixpenny fee as of the advertising club. It might be asserted that a professional man could not live on a sixpenny fee unless he gave second-rate service and inferior drugs. Endless rolls of evidence might be brought to show that it would be better for the sick poor to go straight to hospital than to place themselves in the hands of such a "cheap doctor," that such doctoring was a sham and a pretence, that the whole business was a degrading immorality. A very good case could be made out, and it might prove easy to persuade the Council that it was "infamous in a professional respect" to announce one's readiness to give "advice and medicine" for sixpence. The matter as thus put is not entirely an exaggeration, and we are quite sure that the time is coming when a very strict line will have to be drawn between the subjects on which we may properly combine, and those as to which combination tends to drift into the worst form of trades unionism.

